# Umbilical hernia in a patient with refractory ascites and uncontrolled hypothyroidism: a case report

**DOI:** 10.1093/jscr/rjag702

**Published:** 2026-08-13

**Authors:** Anish Sah, Manish Kumar Sah, Apil Sapkota, Sadikshya Ranabhat, Chandana Neupane, Shubham Kumar Thakur

**Affiliations:** Maharajgunj Medical Campus, Institute of Medicine, Tribhuvan University, Maharajgunj Road, Maharajgunj, Kathmandu 44600, Nepal; Manipal College of Medical Sciences, Kathamandu University, Pokhara 33700 Nepal; Manipal College of Medical Sciences, Kathamandu University, Pokhara 33700 Nepal; Manipal College of Medical Sciences, Kathamandu University, Pokhara 33700 Nepal; Manipal College of Medical Sciences, Kathamandu University, Pokhara 33700 Nepal; Maharajgunj Medical Campus, Institute of Medicine, Tribhuvan University, Maharajgunj Road, Maharajgunj, Kathmandu 44600, Nepal

**Keywords:** ascites, umbilical hernia, liver cirrhosis, hernia repair

## Abstract

Umbilical hernias are a common complication in patients with ascites, particularly those with chronic liver disease. Increased intra-abdominal pressure and weakening of the abdominal wall predispose to hernia formation and associated complications. Endocrine disorders such as hypothyroidism may further exacerbate fluid retention and impair tissue integrity. We report a case of a 52-year-old male with recurrent ascites and poorly controlled hypothyroidism who developed a progressively enlarging umbilical hernia requiring surgical repair. This case highlights the importance of early recognition, optimization of comorbid conditions, and timely surgical intervention in high-risk patients.

## Introduction

Umbilical hernias are a frequent complication in patients with ascites, particularly in those with chronic liver disease. The accumulation of fluid within the peritoneal cavity increases intra-abdominal pressure, weakening the abdominal wall and predisposing to hernia formation. Studies report that ~10%–20% of patients with cirrhosis and ascites develop umbilical hernias, with rates rising to 25%–40% in those with refractory or recurrent ascites [1, 2]. These hernias are associated with significant complications, including incarceration, strangulation, spontaneous rupture, and peritonitis. Mortality rates following emergent repair in patients with decompensated cirrhosis have been reported to be as high as 10%–20% [1, 2].

Although chronic liver disease remains the most common cause of ascites, endocrine disorders such as hypothyroidism may also contribute. Poorly controlled hypothyroidism can lead to ascites through mechanisms including increased capillary permeability, impaired lymphatic drainage, and reduced renal free water clearance [3]. Additionally, hypothyroidism is associated with myopathy and reduced connective tissue strength, which may predispose to abdominal wall hernias and impaired wound healing [4].

Surgical management of umbilical hernias in patients with ascites remains challenging. Elective repair in optimized patients has lower morbidity and recurrence rates compared to emergency surgery, which is associated with significantly higher complications and mortality [5]. Early identification and timely intervention following adequate medical optimization are therefore essential.

## Case presentation

**Figure 1:**
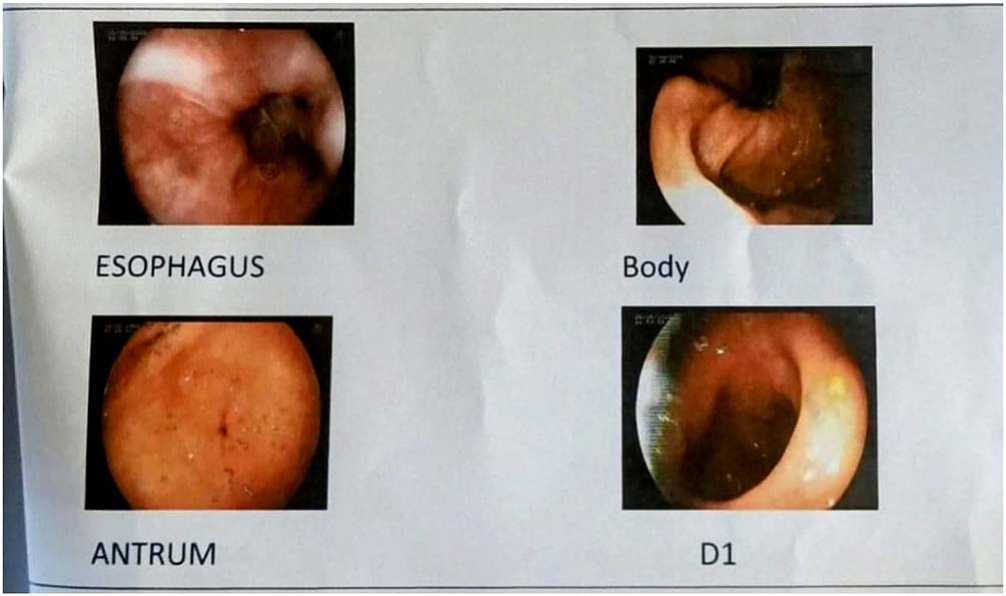
Upper gastrointestinal endoscopy revealed mild esophageal varices.

A 52-year-old male presented to the emergency department with a single episode of hematemesis. He had a long-standing history of chronic alcohol use and multiple prior hospitalizations for recurrent ascites. On admission, he was managed with octreotide, proton pump inhibitors, ceftriaxone, ornidazole, and ondansetron. Urgent upper gastrointestinal endoscopy revealed mild esophageal varices as shown in Fig. 1. Ultrasound (USG) of the abdomen demonstrated moderate ascites along with a newly identified umbilical defect measuring 9 mm. The patient also had a known history of hypothyroidism but was noncompliant with levothyroxine therapy. He was admitted to the intensive care unit for 3 days, during which diagnostic and therapeutic paracentesis was performed. Ascitic fluid analysis showed no evidence of infection or malignancy.

Following stabilization, he was discharged with counseling regarding warning signs of hernia complications. He was strongly advised to abstain from alcohol and smoking and to maintain compliance with levothyroxine therapy.

Two months later, he returned with acute abdominal pain and distension. Repeat USG demonstrated progression of the umbilical defect from 9 to 11 mm. Laboratory evaluation revealed elevated thyroid-stimulating hormone levels, indicating poorly controlled hypothyroidism as shown in Table 1. INR is I.18 and hepatic encephalopathy is not mentioned in radiological test so total Child-Pugh score is 7 putting it in class B. Clinical examination and imaging raised concern for obstruction and possible strangulation. Given the progression of the hernia and worsening symptoms, the patient was advised to undergo surgical repair. After appropriate preoperative optimization, including ascitic drainage, laparoscopic intraperitoneal onlay mesh repair was performed. Pre-operative optimization include paracentesis which was performed 36 hours before surgery, albumin replacement, continuation of diuretic therapy, and optimization of thyroid function with levothyroxine. Electrolyte abnormalities and coagulation parameters were assessed and corrected as required.

**Table 1:** Haematological and biochemical evaluation of patient

Test	Result	Units	Flag	Reference range
**HEMATOLOGICAL TESTS**				
Total WBC count	4200	/cumm	N	4000–11 000
Differential WBC count				
Neutrophils	69	%	N	40–70
Lymphocyte	23	%	N	20–45
Monocytes	04	%	N	2–10
Eosinophils	04	%	N	1–6
Basophils	00	%	N	<2
Haemoglobin	12.6	g/dl	L	13–18
Packed cell volume/hematocrit	38.8	g/dl	L	40–52
Total RBC count	4.30	millions/cumm	L	4.5–6.2
Mean corpuscular volume	90.2	fl	N	92 ± 9
Mean corpuscular hemoglobin	29.3	pg	N	29.5 ± 2.5
Mean corpuscular hemoglobin concentration	32.4	%	N	34 ± 3.0
Platelets	143 000	/cumm	L	150 000–400 000
**BIOCHEMICAL TESTS**				
Renal function tests				
Creatinine	0.9	mg/dl	N	0.6–1.5
Urea	30	mg/dl	N	15–40
Sodium	139.6	mmol/L	N	135.0–150.0
Potassium	4.20	mmolL	N	3.5–5.5
Liver function tests				
Total protein	7.29	g/dl	N	6.0–8.5
Albumin	4.21	g/dl	N	3.2–5.5
Globulin	3.08	g/dl	N	2.5–3.3
A:G ratio	1.36		N	1.0–1.8
Total bilirubin	1.60	mg/dl	H	0.4–1.0
Direct bilirubin	0.56	mg/dl	H	0.1–0.4
Aspartate aminotransferase	33	U/L	N	<40
Alanine aminotransferase	25	U/L	N	<40
Alaline phosphatase	95	U/L	N	41–137

The patient had an uneventful postoperative course, was monitored in the surgical ICU for 1 day, and was discharged in stable condition. On follow-up, he remained compliant with levothyroxine therapy and showed no evidence of recurrence or complications.

## Discussion

Management of umbilical hernias in patients with decompensated chronic liver disease remains complex and controversial. Determining the optimal timing and type of surgical intervention is challenging due to high risks of morbidity, mortality, and recurrence. Historically, a conservative ‘wait-and-watch’ approach was often adopted; however, this strategy is associated with increased risk of complications such as rupture, evisceration, peritonitis, strangulation, and bowel obstruction, frequently necessitating emergency surgery with poorer outcomes [6].

Effective management of ascites plays a pivotal role in improving surgical outcomes. Initial therapy includes sodium restriction and diuretics, while refractory cases may require repeated large-volume paracentesis with albumin supplementation or consideration of transjugular intrahepatic portosystemic shunt (TIPS). Preoperative or intraoperative measures, such as ascitic drainage or shunting procedures, have also been utilized to reduce postoperative complications. Current evidence supports elective or semi-elective repair after adequate medical optimization, which significantly reduces morbidity and mortality compared to emergency surgery [7].

Regarding surgical approach, laparoscopic repair has been associated with lower postoperative morbidity, shorter hospital stays, and faster recovery compared to open repair in selected patients [8, 9]. Additionally, the use of mesh has been shown to reduce recurrence rates compared to primary suture repair, although concerns regarding infection risk remain, particularly in patients with ascites [10, 11]. However We do not advocate routine elective repair for all patients with cirrhosis and ascites. Rather, management should be individualized according to liver disease severity, degree of ascites control, symptom burden, and overall operative risk. Elective repair appears most beneficial in carefully selected patients with adequately optimized ascites and acceptable hepatic reserve, whereas those with advanced decompensation may require further optimization or alternative strategies.

In this case, the presence of uncontrolled hypothyroidism likely contributed to fluid retention and weakening of the abdominal wall, accelerating hernia progression. This highlights the importance of addressing endocrine comorbidities alongside liver disease in such patients.

## Conclusion

Umbilical hernia is a common ventral wall defect in patients with refractory ascites secondary to chronic liver disease and may be further exacerbated by uncontrolled hypothyroidism. Surgical repair in this population remains high risk, particularly in the setting of decompensated liver disease.

Preoperative optimization, including effective control of ascites and management of comorbid conditions, is essential for improving outcomes. Elective repair following optimization is increasingly favored over a conservative approach, although management should be individualized.

In our case, laparoscopic mesh repair resulted in a favorable outcome with no recurrence to date. The role of TIPS in preoperative optimization and the optimal choice between mesh and primary repair require further investigation. Additionally, the contribution of thyroid dysfunction to ascites formation and hernia development warrants further study. Larger prospective studies are needed to establish standardized management guidelines for this high-risk population.

## Key clinical message

We should optimize ascites and comorbidities before surgery in cirrhotic patients with umbilical hernia. Elective or semi-elective repair—preferably laparoscopic with mesh in selected cases—offers better outcomes than emergency surgery. Early intervention after stabilization reduces morbidity, mortality, and recurrence compared to a conservative ‘wait-and-watch’ approach.
